# Searching beyond the Lamppost to Reduce Breast Cancer Disparities

**DOI:** 10.3390/ijerph18031186

**Published:** 2021-01-29

**Authors:** Sarah Gehlert, Marion H. E. Kavanaugh-Lynch, Senaida Fernandez Poole

**Affiliations:** 1 Suzanne Dworak-Peck School of Social Work, University of Southern California, Los Angeles, CA 90089, USA; 2 California Breast Cancer Research Program, Oakland, CA 94612, USA; Marion.Kavanaugh-Lynch@ucop.edu (M.H.E.K.-L.); senaida.poole@ucop.edu (S.F.P.)

**Keywords:** breast cancer, disparities, social determinants, socioeconomic status, health, race, ethnicity

## Abstract

Racial and ethnic differences in breast cancer occur by race/ethnicity in both incidence and mortality rates. Women of lower socioeconomic status likewise have poorer outcomes. When race alone is considered, incidence rates in the United States are highest among White women (130.8 per 100,000), with Black women close behind (126.7 per 100,000). Incidence is lowest among Asian/Pacific Islander women, at 93.2 per 100,000. Mortality differences are more pronounced, with Black women 40% more likely to die from breast cancer than White women (28.4 per 100,000 and 20.3 per 100,000, respectively). Mortality rates for Asian/Pacific Islander women (11.5 per 100,000) are far lower than for Black and White women. When age is considered, additional differences between Black and White women appear, in part accounted for by types of breast cancer experienced. Women of other racial/ethnic groups and socioeconomic status have received less scientific attention. In this article, we provide a brief overview of the evidence for social determinants of breast cancer and argue that the current reliance on race over racism and ethnicity contributes to our inability to eliminate breast cancer disparities in the United States and elsewhere in the world. We suggest alternatives to the current approach to research in breast cancer disparities.

## 1. Introduction

The term “cancer” covers a number of diseases that share common features, principally abnormal cell proliferation, yet differ in expression and outcomes. Breast cancer, for example, has higher rates of survival than most other cancers, yet affects a high number of women. While the five-year survival rate for 2010–2016 was 90%, compared to 10% for pancreatic cancer, the National Cancer Institute estimates that 12.9% of women, or one in eight women, will be diagnosed with breast cancer at some point in their lifetimes [1]. Despite its relatively higher rates of survival, breast cancer remains the second cause of cancer death among women, exceeded only by lung cancer [2]. It is also important to note that long rates of survival mean that a woman with breast cancer can live with it for decades.

Racial and ethnic differences in breast cancer occur in incidence and mortality. The National Cancer Institute’s Surveillance, Epidemiology, and End Results (SEER) program and Center for Disease Control’s National Program of Cancer Registries have followed incidence and survival trends according to Black and White race since 1975, with Asian/Pacific Islander, Hispanic/Latina, American Indian/Alaska Native subpopulations added in 1990.

Marked differences occur by race/ethnicity in both breast cancer incidence and mortality rates. DeSantis et al. analyzed incidence data from 2012 to 2016 and mortality data from 2013 to 2017 and found that incidence rates were highest among White women (130.8 per 100,000), with Black women close behind (126.7 per 100,000) [3]. The two groups are essentially identical in terms of incidence. Incidence was lowest among Asian/Pacific Islander women, at 93.2 per 100,000. Mortality differences are more pronounced, with Black women 40% more likely to die from breast cancer than White women (28.4 per 100,000 and 20.3 per 100,000, respectively). Mortality rates for Asian/Pacific Islander women (11.5 per 100,000) are far lower than for either Black or White women.

## 2. State of the Literature

### 2.1. Multi-Level Contributors to Breast Cancer Disparities

When age is considered, additional differences between Black and White women emerge. Black and White differences in mortality are most pronounced at younger ages and converge with age. Black women 50 years of age and younger, for example, are 1.9–2.6 times more likely to die from breast cancer than White women, while only 1.1–1.2 times more likely to die from the disease at 70 years of age and older [3]. Rates of breast cancer have been increasing in older women, yet studies have yet to be conducted to allow comparison by race [4].

Differences by age can, to some extent, be accounted for by differences in the proportion of breast cancer molecular subtypes, which vary in terms of mortality [5]. Hormone receptor-positive, human epidermal growth factor receptor 2 negative (HR-positive/HER2-negative) breast cancers, which have the most favorable outcomes, are 23% higher in White women over the age of 20 years old than Black women of the same age, and 45% higher than in Hispanic and American Indian/Alaska Native women of the same ages. The triple-negative breast cancer (estrogen receptor-negative (ER-negative), progesterone receptor-negative (PR-negative), and HER2-negative) subtype, which has the least favorable outcomes, is more common among Black women 50 years of age and younger [6].

### 2.2. Social Determinants of Breast Cancer Disparities

Differences in breast cancer incidence and mortality rates are thought to be attributable to a constellation of biological and genetic and social determinants that interact with one another and express themselves differently across racial and ethnic groups. Epigenetic changes occur through changes in how genes are expressed rather than through changes in the underlying DNA sequence [7]. These are more likely to occur during development, especially during what is called “windows of susceptibility,” which are times of heightened developmental change in females [8]. Hilakivi-Clarke et al. suggest that exposure in utero can contribute to intergenerational transmission [9,10].

More distal changes, or changes that do not directly affect either gene expression or cell development, disproportionately affect groups of underrepresented minority women and women of lower socioeconomic status and come about from interacting levels of social factors [11,12]. These interacting factors occur from all levels of influence, from individual factors to neighborhood and community-level factors, social relationships and networks, the availability and quality of institutional resources, and social conditions and policy [13].

Social determinants include a wide range of factors, from smaller social network factors (e.g., extended families) to societal norms, such as housing and educational policy. The World Health Organization defines social determinants as “the conditions in which people are born, grow, live, work, and age” [14]. It further indicates that these are shaped by the distribution of money, power, and resources across the life course. This life-course perspective is important to understanding the development of breast cancer because social and chemical exposures that occur early in life might accumulate to produce breast cancer in adulthood [15].

Coughlin completed an extensive literature review of the social determinants of breast cancer and found convincing evidence for a number of contributors to risk and survival [11]. Lower socioeconomic status has been associated with the likelihood of having more aggressive subtypes, such as HR-positive/HER2-negative breast cancers that occur at a younger age, have poorer survival, and are diagnosed at a later, for example [16,17]. Other contributors include census tract-level poverty, which is associated with late-stage diagnosis and healthcare-related factors, such as health insurance status and stage at diagnosis and survival [18,19]. Late-stage diagnosis is significant because it delays the onset of treatment, thus contributing to mortality.

While breast cancer screening is known to reduce inequalities in breast cancer mortality [20], social barriers to screening such as health insurance and access to care vary by race and ethnicity. The 25 January 2021 Morbidity and Mortality Weekly Report (MMWR) from the Centers for Disease Control and Prevention (CDC) addressed cancer screening receipt in the United States, using 2018 data from 5,311,000 women. The report concluded that the breast cancer screening rates for Black and White women eligible for screening who received screening mammograms were not statistically significantly different (72.9 (67.8–77.6) and 72.7 (71.0–74.3), respectively) [21]. That is to say that no significant differences in screening mammography rates were found when race alone was considered. Differences were attributable to social factors. Lower screening mammography receipt was associated with having lower educational attainment and income, not having a usual source of care, and being uninsured or having only public health coverage. As an example, only 58.6% of women ≤ 138% of the federal poverty threshold compared to 72.1% of women at >250–400% of the federal poverty threshold who were eligible for screening had been screened.

Le Blanc et al. conducted a study on the effect of Medicaid expansion in the United States on rates of late-stage breast cancer diagnosis, and ultimately, on the number of women diagnosed with breast cancer with no insurance [22]. The authors found that the association was particularly striking in Black women, in whom the incidence of advanced disease decreased from 24.6% to 21.6% in expansion states and increased slightly in states that did not expand Medicaid (27.4% to 27.5%). According to the National Center for Health Statistics [23], 13.6% of uninsured persons in the United States were non-Hispanic Black in 2017 and 8.4% were non-Hispanic White.

Adverse life experiences have been implicated in breast cancer disparities. Particularly for women who have lived in adverse social conditions from a young age, continuous exposure in the face of limited mitigating resources such as places in which residents feel safe to interact with others and high-quality health care lead to weathering of the hypothalamic-pituitary-adrenal axis. This occurs because the body must continuously respond to threats and other environmental stressors that take their toll and affect the reproductive hormone system. Linnenbringer et al. suggest that this may contribute to the expression of breast cancer subtypes with less favorable outcomes [24].

In summary, measures of socioeconomic status have been associated with lower survival. The routes through which this occurs might be the late stage at the time of diagnosis and weathering of physiological stress hormone responses through chronic exposure to stressors. Diagnosis at late stages, which may come when women live in areas with fewer public health services that might have provided messaging about cancer risk, fewer breast cancer specialty services, or long waits to see a physician, means that breast cancer may have metastasized, making treatment challenging. Likewise, exposure to chronic stressors predisposes to more aggressive cancers. Both phenomena help explain the disproportionately higher rates of breast cancer mortality seen among underrepresented minority women.

## 3. Key Gaps

Our knowledge of how factors in the social environment interact with biology and genetics to create marked disparities in breast cancer by race and ethnicity and socioeconomic status has been hampered by several factors. First, we lack data that are sufficiently nuanced to understand these interactions. Less research funding has been allocated to social and clinical research than to biomedical research and even less has been allocated to community-based participatory research that includes communities in the planning and execution of research about them. This means that much research too often is designed by breast cancer investigators who are seldom privy to the realities of the women whose lives they are studying. Optimally, more traditional epidemiological studies would be coordinated with community-based participatory research to allow integrated hypothesis generation and testing that is informed by community voices that represent the constellation of underrepresented minority and lower socioeconomic status women who have borne a disproportionate burden from breast cancer for countless decades. More junior investigators will need support to conduct community-based participatory research, which takes time to launch, and otherwise might cause them to be concerned about delaying their preparation for tenure and promotion. Changing university policy to acknowledge differences in data collection would allow the inclusion of community voices.

A second major impediment to our ability to understand breast cancer disparities comes from limitations of how race is considered in research. In trying to make the factors that contribute to disparities measurable, funders such as the National Cancer Institute have restricted the terms of research to standard categories of race. These collapse groups such as Asian American/Pacific Islanders that are as disparate in life experiences as the Hmong of highland Laos and wealthy urban Japanese, and the Hispanic/Latino category, which is constructed to represent groups as unique as rural Mexicans in Baja California and upper-income urban Brazilians. Likewise, because the effects of racism have more to do with disparities than does race itself, the current conceptualizations of race fail to capture the effects of what it means to be an underrepresented minority group member in the United States. This is especially true for Black Americans and American Indians who have experienced countless decades of historical racism. Underrepresented racial and ethnic minority group members, in general, face challenges from racism. Recent attention has focused on stereotypes about Asian Americans [25]. Investigators should work closely with communities experiencing racism to help define it in a way that captures the social realities and life experiences of those experiencing racism. Using community-based participatory research, measurement specialists (e.g., psychometricians) can partner with community members to define racism in terms that allow it to be measured and in ways that allow its inclusion in disparities research. At the same time, investigators and community stakeholders should advocate for change in the way that key variables and measures are used in disparities research.

Another negative outcome from the current reliance on race in research is that it diverts attention from ethnicity-specific trends. Davis Lynn et al. (2018) used National Cancer Institute Surveillance, Epidemiology, and End Results (SEER) data to examine trends for non-Hispanic and Hispanic women. They found that while Black and White women’s incidence rates did converge around 2012, as had previously been reported, the picture changed when ethnicity was considered. In their analysis, incidence rates were highest for non-Hispanic White women and lowest for Hispanic White women, with non-Hispanic Black women in between the two [26]. Thus, future research needs to include ethnicity in group comparisons [26].

Research aimed at eliminating cancer and other disparities is dependent on moving from checking boxes on race, which sanitizes the reality of racism, to an approach that captures the social realities and life experiences of those who have been racialized. Such an approach will move us from abstract research language to research that includes the voices of those being studied, especially those who traditionally have had little or no power. In short, we need to move those who have been on the margins to the center of research.

Eliminating breast cancer and other health disparities ultimately depends on our ability to address policies at the local, state, national, and international levels. Because racism at all levels is systemic, policy change is needed in many sectors (housing, employment, health care, policing, education) that intersect to disadvantage many in society and diminish their lives. This diminution begins at an early age, which is particularly concerning for young females during windows of susceptibility. Lack of equitable funding for schools, for example, produces inequalities. How schools are funded may be our biggest opportunity for changing racial equity. Because inequality accumulates across the life course, starting with children makes sense.

Even if policies are rewritten, however, the social norms will take some time to change. Yet, awareness of their presence is the first step in making change, as is ensuring that policies, once enacted, are followed according to their original intentions. In addition, convincing women to seek treatment from a hospital that they know has treated their loved ones poorly in the past will be a challenge.

Here, we caution against the scientific investigations that generated much of the aforementioned empirical evidence that occurred within the prevailing culture replete with systemic racism. It may, therefore, be vulnerable to structural gaslighting, in which causal relationships are assumed in the absence of scientific support. An example is tacit blaming of women with poor outcomes of breast cancer because they were diagnosed at later stages, under the assumption that it was they who were responsible for their fate rather than a system that makes it markedly different for underrepresented minority and rural women to obtain quality health care.

In fact, Li et al. found that Black women are even more compliant with regard to breast cancer screening than White women [27]. If one replaces the term “late stage” with “advanced stage” or “higher stage” and combines that with data indicating that breast cancer in Black women often progresses more rapidly than in White women, it bolsters our argument that research studies have failed to sufficiently take the experiences of underrepresented minority women and women of lower socioeconomic status into account. The result is that the screening recommendations derived from such studies do not serve all women equitably [28].

In the words of Crawford-Roberts et al:

*Racism, after all, thrives when blame for its outcomes are misattributed. When Black families are refused loans in criminally discriminatory housing schemes, their credit is blamed. When youth of color are disproportionately stopped and frisked, they are told the process is random, and for their safety. And when Black people are killed by police, their character and even their anatomy is turned into justification for their killer’s exoneration [29].*

## 4. Ideas for Future Research

To eliminate racism, research is needed that brings the voices of those individuals and communities who are experiencing racism on a daily basis. Studies that include community stakeholders as equal partners have a much better chance of success and of leading to effective interventions.

More research is needed to understand how the contributors to breast cancer outcomes, which largely come from epidemiological studies, fit together to form a picture within and across underrepresented minority groups, including immigrant and refugee women, and how race/ethnicity and socioeconomic status interact to disadvantage women.

Continued research at the policy level is also important to test the effect of proposed health and other policy changes on underrepresented minority groups and persons of lower socioeconomic status. Krieger et al. explored the association between birth in a so-called Jim Crow state and breast cancer outcomes. The authors found increased odds of ER-breast cancer among Black, but not White, women who were born in these states compared to women born in other states. The effect was most pronounced among women born prior to 1965 [30].

Yet, legislation can improve outcomes. Recent work by Toyoda et al. compared rates of screening mammography among women of lower socioeconomic status in states that did and did not expand Medicaid [31]. The authors found that the screening rates of women ages 50 to 74 years old who made less than USD 15,000 per year went from 62.6% in 2010 (prior to the passage of the Affordable Care Act) to 73.8% in 2018 in expansion states, compared to 68.2% in 2010 to 69.3% in 2018 in non-expansion states. This research helps make clear that policy change aimed at improving the lives of women of lower-socioeconomic status can contribute to improved outcomes.

Clearly, preventing breast cancer depends on understanding the determinants of disparities and targeting them to eliminate disparities by race, ethnicity, and socioeconomic status. Research in partnership with community stakeholders that uses a variety of methods, if integrated, has the potential to decrease breast cancer disparities and improve the quality of life for all.

The onset of the SARS-CoV-19 pandemic further clarifies the urgency of reducing breast and other cancer disparities. Without a doubt, the pandemic will worsen existing disparities in breast cancer. Newman et al. draw attention to the similarities in risk for the SARS-CoV-19 virus and cancer [32], noting that the pattern of disparities apparent in the pandemic is consistent with those observed for cancer. To address both, the authors suggest collaborative strategies that include engaging communities in clinical trials, providing insurance for those who lose employment, and offering additional support for safety-net and public hospitals. We would do well to heed their advice.

## Data Availability

No new data were created or analyzed in this study. Data sharing is not applicable to this article.
